# Prevalence and prognosis impact of right ventricular injury in patients with acute respiratory distress syndrome: a systematic review and meta-analysis

**DOI:** 10.1016/j.aicoj.2026.100117

**Published:** 2026-07-16

**Authors:** Mathieu Jozwiak, Adrien Joseph, Alan Mourougayen, Matthieu Petit, Antoine Vieillard-Baron, Philippe Vignon, Bruno Evrard

**Affiliations:** a Service de Médecine Intensive Réanimation, CHU de Nice, Nice, France; b UR2CA, Equipe CARRES, Université Côte d'Azur, Nice, France; c Medical and Surgical ICU, University Hospital Ambroise Pare, AP-HP, Boulogne-Billancourt, France; d UMR 1018, CESP, Université Versailles Saint Quentin en Yveline, France; e Medical Surgical Intensive Care Unit, Limoges University Hospital, Limoges, France; f CIC 1435-INSERM, Limoges, France; g University of Limoges, Limoges, France

**Keywords:** Acute cor pulmonale, Acute respiratory distress syndrome, Echocardiography, Mortality, RV injury

## Abstract

**Background:**

Right ventricular (RV) injury is a frequent complication in patients with acute respiratory distress syndrome (ARDS), yet its prevalence and prognostic significance remain uncertain. The main objective of this systematic review and meta-analysis was to evaluate the association between RV injury and mortality in patients with ARDS. The secondary objectives were to determine the prevalence of RV injury and to assess the impact of both COVID-19 status and the definition of RV injury on mortality.

**Methods:**

From January 2013 to December 2024, we searched MEDLINE, EMBASE and the Cochrane Central Register of Controlled Trials for all studies including adult patients with ARDS according to the Berlin definition, which reported mortality according to the presence or absence of RV injury.

**Results:**

Twenty-three studies including 3,977 patients with ARDS were analyzed. The pooled prevalence of RV injury was 30% (95% confidence intervals (CI) 23–38%), ranging from 6% to 56% across studies depending on the definition used, and did not differ between COVID-19 (33%, 95%CI 22–47%) and non-COVID-19 (25%, 95%CI 21–29 %) patients. RV injury was associated with increased ICU mortality in unadjusted analyses (random-effects odds ratio (OR) 2.49, 95%CI 1.81–3.42; I^2^ = 56%) and in adjusted analyses (aOR 2.37, 95%CI 1.24–4.51, I^2^ = 42%; adjusted hazard ratio (aHR) 2.64, 95%CI 1.60–4.37, I^2^ = 24%). This association remained consistent across subgroups based on COVID-19 status and RV injury definition, and across sensitivity analyses excluding patients receiving venovenous extracorporeal membrane oxygenation and low to moderate quality studies.

**Conclusions:**

RV injury is common in patients with ARDS and is consistently associated with increased mortality, irrespective of the definition used. Further studies enrolling consecutive patients, with standardized and repeated echocardiographic assessment of RV function, are needed to establish causal inference.

**Trial registration:**

CRD42024568063 and date of registration: 22/07/2024.

## Graphical abstract

**Figure:**
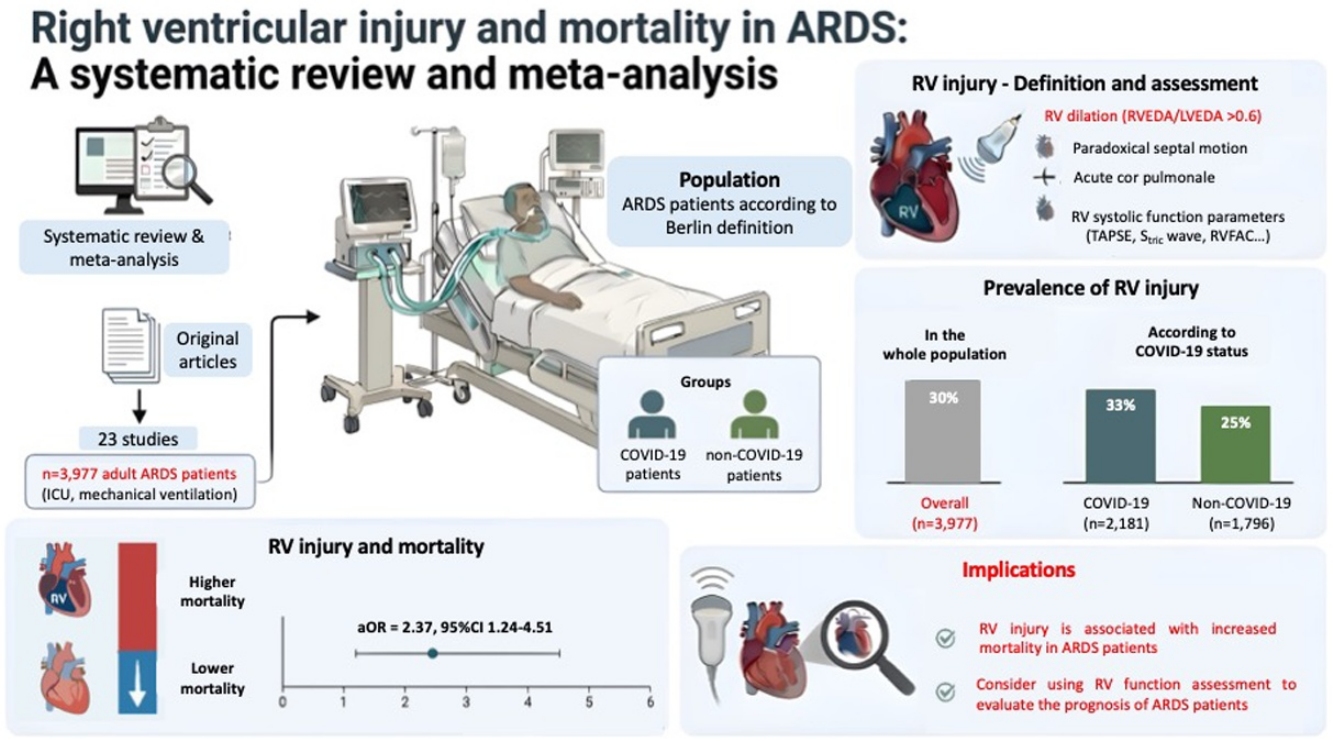

## Background

Acute respiratory distress syndrome (ARDS) accounts for approximately 10% of admission in intensive care unit (ICU) and is associated with significant morbidity and mortality, the mortality rate ranging up to 45% in severe ARDS [1,2].

Right ventricular (RV) injury is a frequent complication in patients with ARDS, with a prevalence ranging from 22 to 50%, depending on the definition used [3]. Currently, RV injury in patients with ARDS is defined as “*a clinical syndrome with a spectrum of abnormal RV biomechanics encompassing RV dilatation, RV dysfunction, RV failure or a combination thereof with or without circulatory (arterial and/or venous) and other organ system consequence*” [4]. The pathophysiology of RV injury is multifactorial, involving increased RV afterload secondary to hypoxic pulmonary vasoconstriction, hypercapnia, pulmonary vascular remodelling, and the effects of positive pressure ventilation on pulmonary vascular resistance [5,6]. Despite protective ventilation, approximately 20% of patients with ARDS still develop acute cor pulmonale (ACP) during their ICU stay, which is associated with a higher mortality rate [7,8].

Through ventricular interdependence, RV dilation can impair left ventricular filling and reduce cardiac output, potentially compromising systemic oxygen delivery [5,6]. Previous meta-analyses have suggested an association between RV injury and increased mortality in patients with ARDS [9,10]. However, these analyses included studies conducted before the Berlin definition of ARDS, which standardized diagnostic criteria and stratified disease severity. Moreover, advances in ARDS management, including protective ventilation strategies, prone positioning, and extracorporeal support, may have modified the incidence and clinical impact of RV injury. Furthermore, the definitions of RV injury varied considerably across studies, ranging from isolated RV dilation to more comprehensive assessments including systolic function and the presence of ACP. Finally, additional studies, including studies with content-standardized echocardiographic follow-up [[11], [12], [13], [14], [15], [16]] and studies in patients with COVID-19 [11,13,14,[16], [17], [18], [19], [20], [21], [22], [23], [24], [25]] have been published since these former meta-analyses, providing new data on the prognostic significance of RV injury in contemporary ARDS management.

The main objective of this systematic review and meta-analysis was to comprehensively evaluate the association between RV injury and mortality in patients with ARDS defined by the Berlin definition. We hypothesized that RV injury would be significantly associated with increased mortality in patients with ARDS. Secondary objectives included the prevalence of RV injury as well as the impact of COVID-19 status and the definition of RV injury on mortality.

## Methods

### Protocol and registration

The protocol of this study was preregistered on PROSPERO (CRD42024568063). This study followed the Preferred Reporting Items for Systematic Reviews and Meta-analyses (PRISMA) reporting guidelines [26] (Table S1).

### PECO question

In adult patients with ARDS according to the Berlin definition (Population), is RV injury (Exposure) compared with absence of RV injury (Comparator) associated with increased mortality (Outcome)?

### Search strategy

MEDLINE via PubMed (including In-Process and Epub ahead of print), EMBASE and the Cochrane Central Register of Controlled Trials databases were systematically searched with restriction to English-language publications from 1 January 2013 to 31 December 2024 to consider only studies including patients with ARDS according to the Berlin definition [27]. Trial registries including ClinicalTrials.gov were also considered to identify completed and ongoing trials. The search strategy combined keywords and Medical Subject Headings (MeSH) terms related to ARDS and RV injury. The full search string for PubMed was: ((((((((acute respiratory distress syndrome[Title/Abstract]) OR (ARDS[Title/Abstract])) AND (right ventricle[Title/Abstract])) OR (right ventricular[Title/Abstract])) OR (right ventricular injury[Title/Abstract])) OR (right ventricular dysfunction[Title/Abstract])) OR (right ventricular failure[Title/Abstract])) OR (acute cor pulmonale[Title/Abstract])) OR (RV injury[Title/Abstract])) OR (RV dysfunction[Title/Abstract])) OR (RV failure[Title/Abstract]). The reference lists of relevant articles and recent systematic reviews were manually searched to identify additional potentially eligible studies using the snowballing method.

### Eligibility criteria

We searched for studies including adult patients with ARDS allowing comparisons of patients with and without RV injury, irrespective of the definition of RV injury used. RV injury was defined as the presence of ACP and/or RV systolic dysfunction and/or RV failure defined as the dilation of the RV with or without central venous pressure (CVP) >8 mmHg. We excluded studies in which mortality was not reported in both groups of patients, studies including spontaneously breathing patients with ARDS, studies published in languages other than English, conference proceedings, letters, case reports and abstracts without full-text availability, animal studies and meta-analyses.

### Study selection and data extraction

Six authors (MJ, AJ, AM, MP, PV and BE) independently screened titles and abstracts of all retrieved records using Rayyan software [28]. Articles potentially meeting inclusion criteria were retrieved in full text and reviewed independently by the same six authors to verify eligibility. Disagreements at any stage were resolved through discussion when necessary.

Five authors (MJ, AJ, AM, MP and BE) independently extracted data using a standardized electronic data extraction form. Discrepancies were resolved by consensus or by a sixth author (PV) when needed. The following data were extracted from each included study: study characteristics, patient characteristics, definition of RV injury, hemodynamic and respiratory variables concomitant to RV function assessment.

### Outcomes

The primary outcome was mortality, including ICU mortality, in-hospital mortality 28-day mortality, 90-day mortality or longest available follow-up.

Secondary outcomes included prevalence of RV injury, mortality in patient with and without COVID-19, and mortality according to the definition of RV injury.

### Quality assessment

A quality assessment was independently performed by two authors (MJ and AJ) at both the individual study level and outcome level, using the using a modified version of the Newcastle-Ottawa quality assessment scale (NOS) [29]. All disagreements were resolved by consensus or consultation with a third author (BE).

The overall quality of evidence for each outcome was assessed using the Grading of Recommendations Assessment, Development and Evaluation (GRADE) approach [30]. This tool rates the overall quality of evidence as high, moderate, low, or very low, based on five domains: risk of bias, inconsistency, indirectness, imprecision and other considerations (publication bias, large effect, plausible confounding, and dose response gradient).

### Statistical analysis

The meta-analysis was conducted according to predefined mortality timepoints (ICU, 28-day, 90-day and in-hospital mortality). When multiple mortality endpoints were reported within the same study, ICU mortality was preferentially selected. Each study was included only once per analysis to avoid duplication of data. Global meta-analyses were then performed with stratification by mortality endpoint. When raw event data were available, unadjusted odds ratios (OR) for mortality were calculated by comparing patients with RV injury to those without RV injury. These estimates were pooled using random-effects Mantel–Haenszel models with a continuity correction of 0.5 applied when necessary. Between-study variance was estimated using the Paule–Mandel estimator, and confidence intervals were computed using the Hartung–Knapp adjustment. When studies reported adjusted effect estimates, adjusted odds ratios (aOR) and adjusted hazard ratios (aHR) were extracted separately along with their corresponding 95% confidence intervals (CIs). The variables of adjustment used in the 23 studies included in the meta-analysis are summarized in Table S2. Adjusted estimates were extracted as reported in each study, no additional adjustment was performed. Adjusted ORs and adjusted HRs were not combined in the same meta-analysis. Effect estimates were analyzed on the logarithmic scale, and standard errors were reconstructed from reported confidence intervals using conventional methods. Pooled estimates were obtained using random-effects inverse-variance models with the Paule–Mandel estimator and Hartung–Knapp adjustment.

The prevalence of RV injury was estimated using a random-effects model with a logit transformation (PLOGIT) to stabilize variances. Between-study variance was estimated using the restricted maximum likelihood (REML) method. Prevalence estimates were stratified according to the definition of RV injury. We also performed sensitivity analyses according to (i) patient COVID-19 status, (ii) after excluding studies with patients requiring VV-ECMO and (iii) after excluding studies with a NOS score <8 (moderate to low quality).

Statistical heterogeneity was assessed using Cochran’s Q test and quantified using the I^2^ statistic. Substantial heterogeneity was predefined as p < 0.10 for the Q test and/or I^2^ >50%. Given the expected clinical and methodological heterogeneity among observational studies, random-effects models were used throughout. Funnel plots were generated if at least 10 studies were available and asymmetry was tested using Egger test. All statistical analyses were performed using R (packages meta). All tests were two-sided, and a p-value <0.05 was considered statistically significant.

## Results

### Study characteristics

**Fig. 1:**
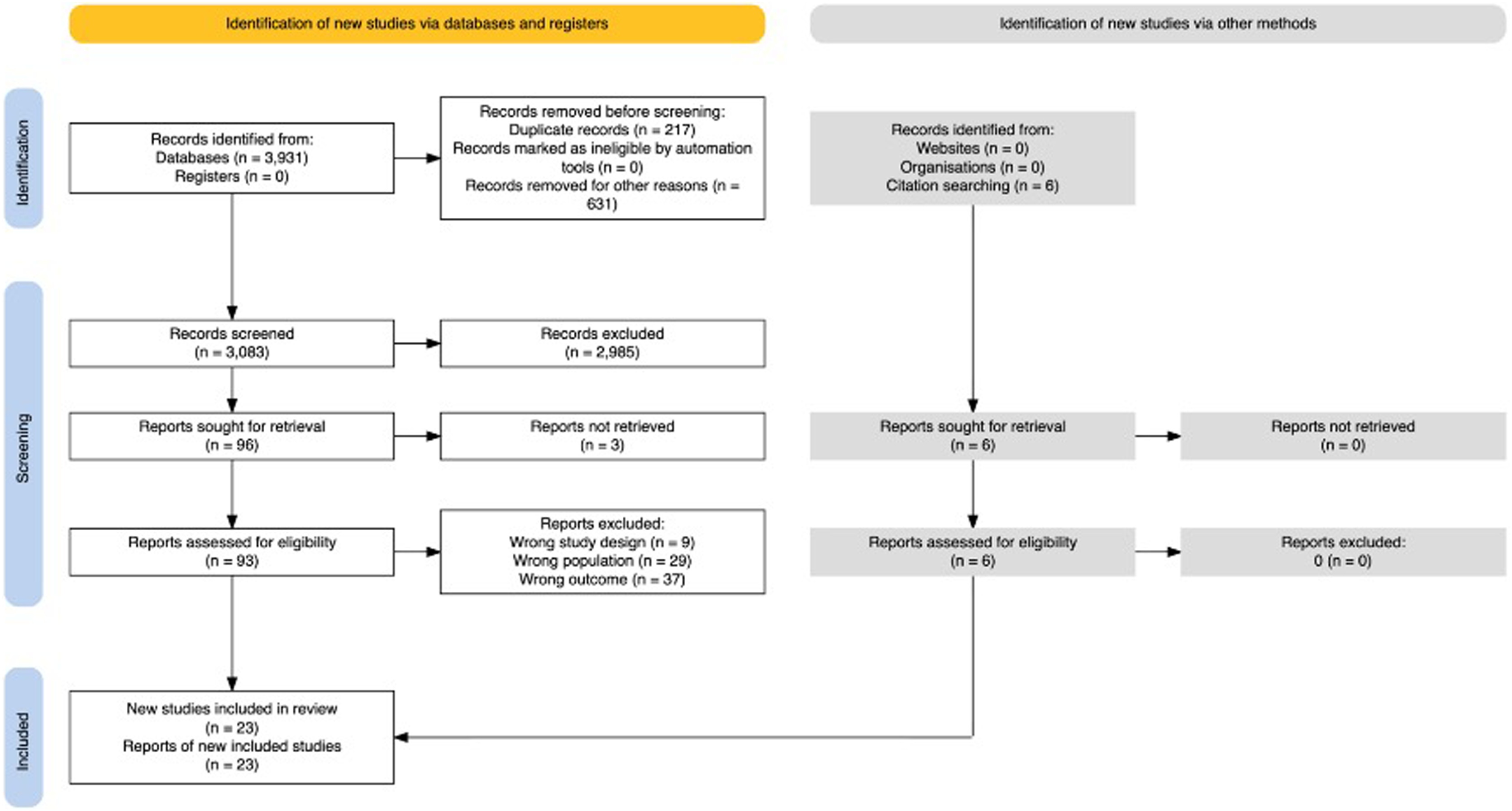
Flowchart of the included studies.

The electronic search identified 3,931 citations of which 93 were retained for full-text assessment. Overall, 23 studies representing 3,977 patients with ARDS were included in the analysis [7,8,[11], [12], [13], [14], [15],[17], [18], [19], [20], [21], [22], [23], [24], [25],[31], [32], [33], [34], [35], [36], [37]] (Fig. 1). Study sample sizes ranged from 33 to 752 patients. Most of the studies were conducted in Europe, 8 were retrospective and 15 were prospective (Table 1). Overall, 14 studies included 2,181 patients with COVID-19 related ARDS, while 9 studies included 1,796 patients with non-COVID-19 ARDS. Data on adjunctive therapies and organ support were inconsistently reported. Prone positioning was reported in 11 studies, renal replacement therapy (RRT) in 8 studies, use of inhaled nitric oxide in 6 studies, and venovenous extracorporeal membrane oxygenation (VV-ECMO) in 7 studies. When reported, the use of prone positioning ranged from 11% to 72%, inhaled nitric oxide from 0% to 62%, VV-ECMO from 0% to 100%, and RRT from 1% to 51% (Table S3).

### Definitions of RV injury

RV injury was defined according to echocardiographic criteria in all studies: 12 used transthoracic echocardiography only, 3 used transoesophageal echocardiography only and 8 used both. None of them used pulmonary artery catheter to define RV injury. A single examination was performed in 19 studies and repeated examinations during ICU stay were performed in 4 studies. The timing of RV evaluation was mentioned in 16 studies: within the first 24 h of ICU admission in 2 studies, within 24−48 h of ICU admission in 5 studies, within 48−72 h of ICU admission in 1 study, within 72 h of ICU admission in 1 study, >72 h of ICU admission in 5 studies, >7 days of ICU admission in 1 study and at multiple predefined timepoints in 1 study (Table 1).

**Table 1:** Characteristics of the 23 studies included in the meta-analysis.

First author	Year	Continent	Study design	Sample size	Study period	ICU setting	RV injury definition	Prevalence of RVI (%)	COVID-19 status	Repeated or single echocardiographic evaluation	Timing of RVI evaluation	Type of echocardiography
Boissier [7]	2013	Europe	Prospective	226	2004−2009	Medical	RVEDA/LVEDA ratio > 0.6 with paradoxical septal motion	22	No	Single	24−48 h	TEE
Lheritier [31]	2013	Europe	Prospective	200	2009−2012	Medical & Surgical	RVEDA/LVEDA ratio > 0.6 with paradoxical septal motion	23	No	Single	24−48 h	Both
Lazzeri [32]	2016	Europe	Prospective	74	2013–2015	Medical	TAPSE < 16 mm OR RVEDA/LVEDA ratio > 0.6	23	No	Single	>3 days	Both
Mekontso-Dessap [8]	2016	Europe	Prospective	752	1994−2012	Medical & Surgical	RVEDA/LVEDA ratio > 0.6 with paradoxical septal motion	22	No	Single	24−48 h	Both
Lazzeri [34]	2018	Europe	Prospective	121	2009−2015	Medical	RVEDA/LVEDA ratio > 0.6	36	No	Single	NA	Both
See [33]	2017	Asia	Prospective	234	2012−2014	Medical	RVEDA/LVEDA ratio > 1 with paradoxical septal motion	28	No	Single	24−48 h	TTE
Lazzeri [35]	2018	Europe	Retrospective	92	2012−2015	Medical	RVEDA/LVEDA ratio > 0.6	33	No	Single	NA	Both
Zeiton [36]	2018	Africa	Prospective	45	2016	Medical & Surgical	RVEDA/LVEDA ratio > 0.6 with paradoxical septal motion	22	No	Single	<72 h	TTE
Chotalia [17]	2021	Europe	Retrospective	172	2020	Medical	Dilation (RV/LVEDA > 0.6) AND/OR Systolic impairment (TAPSE < 17 mm or RV FAC < 35%)	77	Yes	Single	>3 days	TTE
Beyls [18]	2022	Europe	Retrospective	86	2020−2021	Medical	Systolic impairment (RV FAC < 35% or RV-FWLS <−20% or RV LSF < 20%)	43	Yes	Single	48−72 h	TEE
Chotalia [19]	2022	Europe	Retrospective	305	2020−2021	Medical	Dilation (RV/LVEDA > 0.6) AND/OR Systolic impairment (TAPSE < 17 mm or RV FAC < 35%)	17	Yes	Single	>3 days	TTE
Khorsandi [20]	2022	North America	Retrospective	33	2020−2021	Medical	TAPSE < 17 mm or RV FAC < 35%	42	Yes	Single	NA	TTE
McCall [21]	2022	Europe	Prospective	112	2020−2021	Medical	RVEDA/LVEDA ratio > 0.6 with paradoxical septal motion	6	Yes	Single	>3 days	TTE
Valenzuela [22]	2022	South America	Prospective	140	2020	Medical	RVEDA/LVEDA ratio > 0.6 OR RVEDA/LVEDA ratio > 0.6 with paradoxical septal motion	39	Yes	Single	<24 h	TTE
Evrard [12]	2023	Europe	Prospective	182	2020−2021	Medical	Paradoxical septal motion	NA	Yes	Repeated	NA	TEE
Huang [13]	2023	International	Prospective	281	2020−2021	Medical	RVEDA/LVEDA ratio > 0.6 with paradoxical septal motion AND/OR RVEDA/LVEDA ratio > 0.6 and CVP > 8 mmHg or dilated IVC (if CVP unavailable) AND/OR TAPSE < 17 mm	37	Yes	Repeated	NA	Both
Lazzeri [23]	2023	Europe	Prospective	61	2020–2021	Medical	RVEDA/LVEDA > 0.6 and TAPSE < 15 mm	56	Yes	Single	>7 days	TTE
Cain [11]	2024	North America	Retrospective	243	2020	Medical	RV dilation and increased RV pressure or paradoxical septal motion	33	Yes	Repeated	Any time of ICU hospitalization	Both
Cheong [24]	2024	South America	Retrospective	114	2020–2022	Medical	RV dilation (RV basal diameter >41 mm) with or without paradoxical septal motion	12	Yes	Single	<24 h	TTE
Jozwiak [14]	2024	Europe	Prospective	118	2020−2021	Medical	RVEDA/LVEDA ratio > 0.6 with paradoxical septal motion AND/OR Systolic impairment (TAPSE < 17 mm or systolic tricuspid annular velocity < 9.5 cm/s or RV FAC < 35%)	45	Yes	Repeated	Multiple predefined timepoints	TTE
Petit [25]	2024	Europe	Retrospective	158	2020−2021	Medical	RVEDA/LVEDA ratio > 0.8 with paradoxical septal motion	20	Yes	Single	>3 days	Both
Tsolaki [15]	2024	Europe	Prospective	176	2020−2022	Medical	RVEDA/LVEDA ratio > 0.6 with paradoxical septal motion OR Systolic impairment (Two indices among RV FAC < 35%, TAPSE < 16 mm, RVEF < 44%, RVLS > − 20%)	NA	Yes	Repeated	24−48 h	TTE
Xingzheng [37]	2024	Asia	Prospective	52	2020–2022	Medical	RVEDA/LVEDA ratio > 0.6 with paradoxical septal motion	25	No	Single	NA	TTE

CVP: central venous pressure, IVC: inferior vena cava, LVEDA: left ventricular end-diastolic area, NA: non-available, RVEDA: right ventricular end-diastolic area, RVEF: right ventricular ejection fraction, RV FAC: right ventricular fractional area change, RV FWLS: right ventricular longitudinal shortening fraction, RVLS: right ventricular longitudinal strain, TEE: transesophageal echocardiography, TTE: transthoracic echocardiography, TAPSE: tricuspid annular plane systolic excursion.

The definitions of echocardiographic RV injury were highly heterogenous across studies (Table 1). Two studies relied only on a parameter of RV systolic function, whereas 10 studies used the combination of RV dilation and paradoxical septal motion (i.e. acute cor pulmonale). Additional definitions included RV dilation and/or impairment of at least one parameter of RV systolic function in 5 studies, isolated RV dilation in 3 studies, paradoxical septal motion only in 1 study and RV dilation with increased CVP or inferior vena cava dilation in 1 study. Beyond theses definitional differences, the assessment of RV systolic function itself was inconsistent. For instance, tricuspid annular plane systolic excursion was used in 8 studies, the systolic tricuspid annular velocity in 1 study and the RV fractional area change in 6 studies (Table 1).

### Prevalence of RV injury

**Fig. 2:**
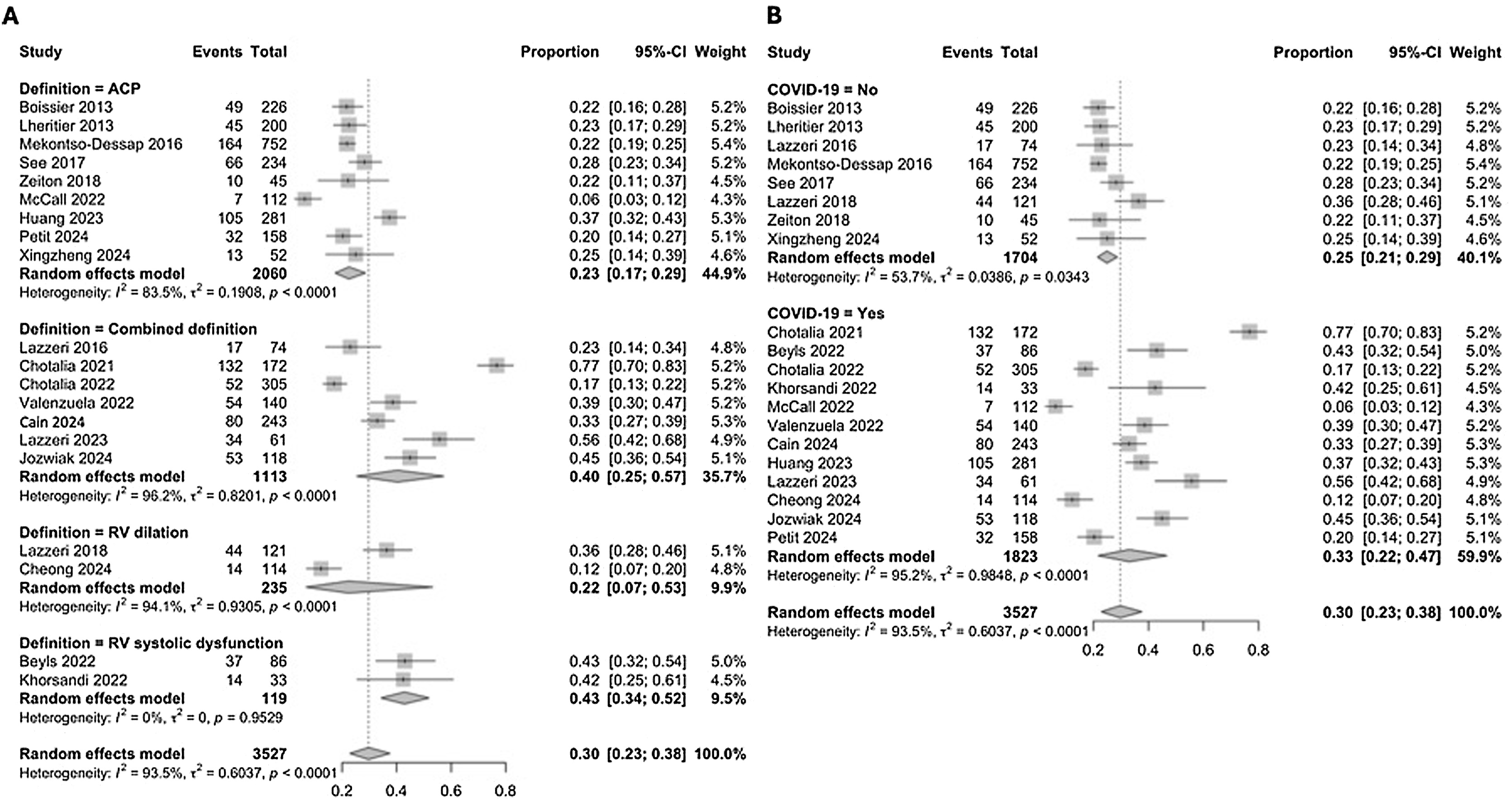
Prevalence of right ventricular (RV) injury according to its definition (panel A) and COVID-19 status of patients (panel B). Combined definition refers to studies in which several criteria have been used to define RV injury.

The prevalence of RV injury ranged from 6% to 56% across studies and varied substantially according to the definition used, with the greatest variability observed among studies using combined echocardiographic definitions (Table 1 and Fig. 2A). Overall, the pooled prevalence of RV injury was 30% (95% CI 23–38%). When stratified by COVID-19 status, the pooled prevalence was 25% (95% CI 21–29%) in non-COVID-19 populations and 33% (95% CI 22–47%) in COVID-19 populations (Fig. 2B).

### RV injury and mortality

**Fig. 3:**
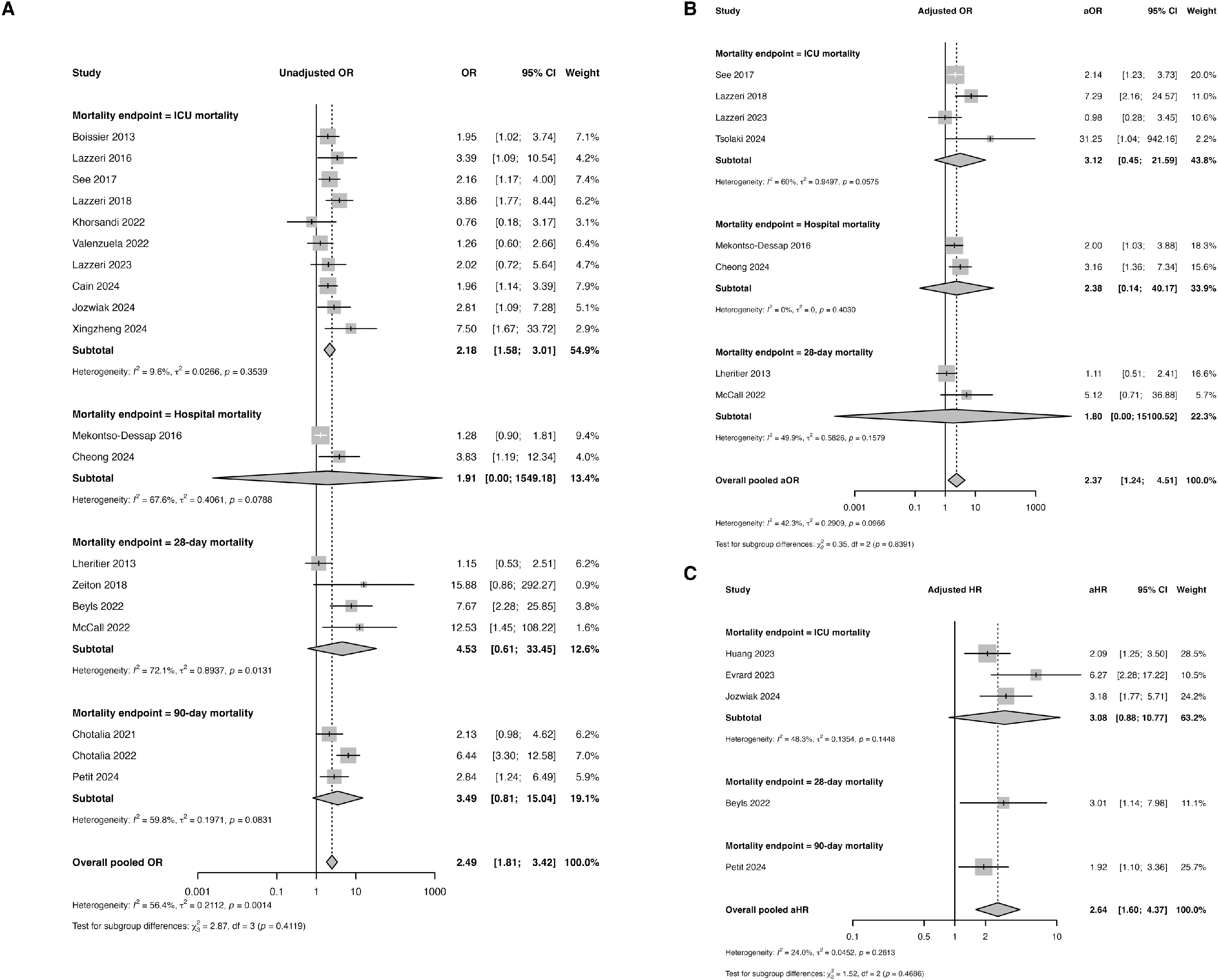
Forest plots for unadjusted (panel A) and adjusted (panels B and C) mortality irrespective of the definition of right ventricular injury. OR: odds ratio, HR: hazard ratio, CI: confidence interval.

ICU mortality was reported in 14 studies, 28-day mortality in 4 studies, 90-day mortality in 3 studies and in-hospital mortality in 2 studies (Table S3). RV injury was associated with mortality at univariate analyses (pooled random-effects OR 2.49, 95%CI 1.81–3.42; I^2^ = 56%) (Fig. 3A). At multivariate analyses, RV injury remained associated with mortality (pooled random-effects aOR 2.37, 95%CI 1.24–4.51, I^2^ = 42% and aHR 2.64, 95%CI 1.60–4.37, I^2^ = 24%) (Fig. 3B and 3C).

**Fig. 4:**
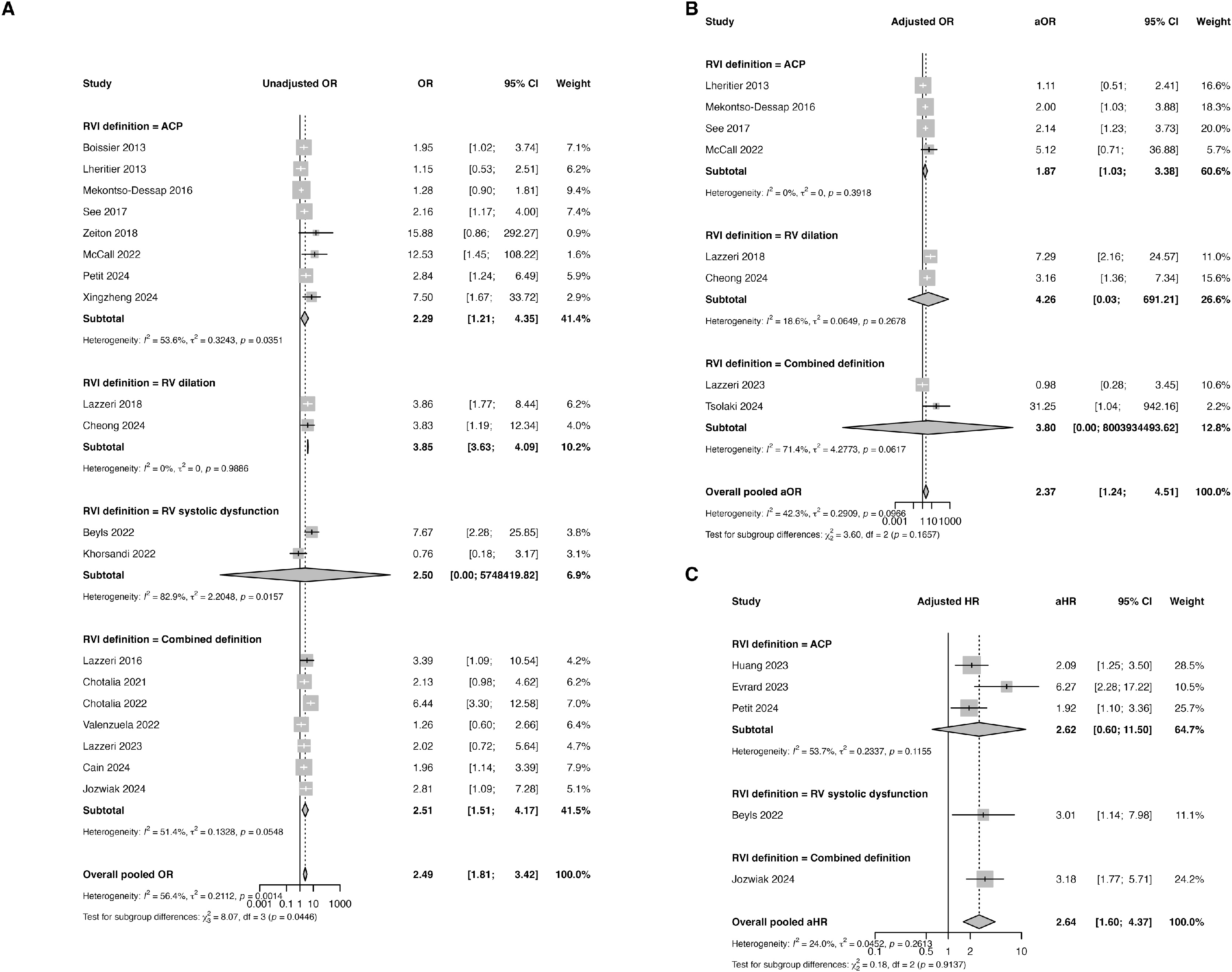
Forest plots for unadjusted (panel A) and adjusted (panels B and C) mortality according to the definition of right ventricular injury. OR: odds ratio, HR: hazard ratio, CI: confidence interval, ACP: acute cor pulmonale, RVI: right ventricular injury.

When stratified according to COVID-19 status, RV injury remained consistently associated with mortality in both unadjusted and adjusted analyses (Figure S1). When exploring the impact of RV injury definitions on mortality (Fig. 4), ACP was associated with mortality in both unadjusted and adjusted analysis (pooled aOR 1.87 95%CI 1.03–3.38, I^2^ = 0%). Similar findings were observed in studies using combined definitions of RV injury. Studies relying on parameters of RV systolic function only were limited (n = 2) and univariable estimates only were available, with no significant association observed.

### Sensitivity analyses

Results remained consistent when excluding studies with patients receiving VV-ECMO (Figure S2). Similar findings were observed when moderate and low-quality studies were excluded (Figure S3).

### Study quality and publication bias

There were 13 (56%) studies of high quality, 5 (22%) studies of moderate quality and 5 (22%) studies of low-quality (Table S4). The GRADE assessment of the certainty of the evidence supporting the association of RV injury with mortality and other outcomes was very low or low (Table S5).

Visual inspection of the funnel plot suggested some degree of asymmetry (Egger’s regression test, p = 0.01), with a dispersion of effect estimates across a wide range of standard errors. This pattern was observed across studies of varying sizes and was not restricted to smaller studies. (Figure S4).

## Discussion

The present systematic review and meta-analysis showed that, among patients with ARDS, the pooled prevalence of RV injury was approximately 30%, and that RV injury was consistently associated with increased mortality. This association remained robust irrespective of COVID-19 status of patients and across sensitivity analyses. While RV injury was defined according to echocardiographic criteria only in all studies, we found that the estimated prevalence of RV injury varied according to the definition used, with higher prevalence observed when combined definitions were applied.

### Association between RV injury and mortality

RV injury was associated with ICU mortality as well as with other mortality endpoints including 28-day, 90-day, and in-hospital mortality. These results are consistent with previous meta-analyses [9,10] and extend them by including a larger number of studies conducted exclusively in the post-Berlin definition era, ensuring greater homogeneity in ARDS diagnosis.

A key methodological issue relates to the heterogeneity of mortality endpoints used across studies [38]. In previous meta-analyses, different mortality timepoints were frequently pooled together into a single outcome, which may increase statistical power but also introduces important clinical heterogeneity. ICU mortality, 28-day mortality, 90-day mortality, and in-hospital mortality do not capture the same dimension of prognosis [39]. ICU mortality is more likely to reflect the acute consequences of respiratory and hemodynamic failure, whereas 28-day or 90-day mortality may additionally capture later complications such as nosocomial infection, persistent multiorgan dysfunction, treatment limitation, or frailty-related deterioration [39,40]. In-hospital mortality may also be influenced by local discharge practices and healthcare organization [41]. For this reason, combining these endpoints is methodologically imperfect and the resulting pooled estimate should be interpreted with caution. In our study, ICU mortality was prioritized when several mortality endpoints were available from the same study to avoid double counting, and analyses were stratified according to mortality endpoint. However, because of the limited number of studies available for several individual timepoints, an overall estimate across reported mortality outcomes was also examined. This overall association should therefore be viewed as a global signal rather than as a precise estimate of a single clinically uniform endpoint.

The relationship between RV injury and mortality was also difficult to estimate precisely in our analysis, because most available studies remain observational, even when adjusted analyses are reported. Since most of the studies included in this meta-analysis did not adjust for confounding factors such as ARDS severity, driving pressure, hemodynamic failure (shock, vasopressors use and dose), venous congestion and organ dysfunction such as acute kidney injury [42], residual confounding is therefore likely. This is particularly important because the occurrence of RV injury in ARDS is dynamic and may evolve over time [[12], [13], [14], [15], [16]], according to changes in lung mechanics, pulmonary vascular load, gas exchange, hemodynamics, and therapeutic interventions. Yet most included studies relied on a single echocardiographic assessment and were therefore unable to capture this temporal dimension. In addition, adjunctive therapies that may substantially modify both RV function and prognosis, such as prone positioning, inhaled nitric oxide, RRT, or VV-ECMO, were inconsistently reported across studies. This is especially relevant for prone positioning, which should be considered standard of care in moderate-to-severe ARDS and may influence both RV afterload [[43], [44], [45]] and outcomes [46]. As a result, RV injury may have been captured at different moments of the disease course and under very different treatment conditions across studies, further compounded by the heterogeneity of the timing of echocardiographic assessment.

From this perspective, longitudinal studies likely provide a more clinically relevant framework for understanding the prognostic significance of RV injury. Studies by Huang [13], Evrard [12,16] and Jozwiak [14] are particularly informative because they account, at least in part, for the evolution of RV injury over time rather than treating it as a fixed baseline phenomenon. This dynamic approach is probably better suited to ARDS pathophysiology, in which RV dysfunction may appear, worsen, or improve according to disease progression and response to therapy.

### Definitions of RV injury

One of the key findings of this review is the marked heterogeneity in the definitions of RV injury across studies. RV injury was defined using a wide range of echocardiographic criteria, from isolated RV dilation [34,35] to definitions combining RV dilation with systolic dysfunction [17,19,23,32] or ACP [7,8,[11], [12], [13], [14], [15],^21^,^22^,^24^,^25^,^31^,^33^,^36^,^37^]. Some studies relied on a single echocardiographic parameter, whereas others combined multiple indices of RV structure and function, and in some cases integrated echocardiographic findings with surrogates of elevated right-sided pressures such as increased CVP or inferior vena cava dilation [13]. Moreover, imaging modality varied across studies, with the use of either transthoracic or transoesophageal echocardiography, which may influence measurement accuracy and reproducibility. Most studies also reported the RV/left ventricular (LV) end-diastolic areas ratio rather than the guideline-recommended linear RV/LV end-diastolic diameters ratio to define RV dilation. Given the triangular RV geometry, this choice was data-driven and future studies should report guideline-concordant linear ratios. This heterogeneity was also observed within definitions that could be considered relatively consensual, such as ACP. Thresholds used to define RV dilation varied across studies, and some definitions focused on more severe forms of RV enlargement, while others applied broader criteria. Furthermore, the assessment of RV dilation is inherently dependent on image quality and operator expertise, which may introduce additional variability compared with more quantitative parameters of RV systolic function.

Overall, this lack of standardization has important implications for the interpretation of associations between RV injury and outcomes. Although we observed a consistent association between RV injury and mortality across definitions, the use of heterogeneous and sometimes overlapping criteria likely contributes to between-study variability and limits the ability to draw firm conclusions regarding the prognostic value of specific echocardiographic patterns. Notably, few studies evaluated isolated parameters of RV systolic function, and these did not consistently demonstrate a significant association with mortality. Moreover, because these parameters were most often incorporated into broader multiparametric definitions of RV injury, their independent prognostic value remains difficult to determine.

Taken together, these findings highlight the urgent need to move toward a more standardized and clinically meaningful definition of RV injury in ARDS. Current recommendations suggest combining several quantitative parameters of RV systolic function [47] and avoiding reliance on visual assessment alone [48], but these approaches have not yet been uniformly adopted in clinical research.

### Prevalence of RV injury in ARDS

The prevalence of RV injury in this meta-analysis showed substantial variability, ranging from 6% to 56%, largely driven by differences in definition and study design. Studies using combined definitions such reported higher prevalence rates, whereas studies defining RV injury as ACP reported lower prevalence rates but a stronger association with mortality. This suggests a spectrum of RV injury in patients with ARDS, ranging from isolated RV dilation to ACP. The timing of echocardiographic assessment also appeared to be a major determinant of reported prevalence. Studies performing a single early echocardiographic evaluation likely captured only a snapshot of RV function, whereas those incorporating repeated assessments reported higher cumulative prevalence of RV injury over time [14,16]. Consequently, standardized and repeated echocardiographic evaluation during ICU stay may provide a more accurate assessment of RV involvement and improve the identification of patients at risk.

### Strengths and limitations

We restricted our analysis to studies conducted after the Berlin definition of ARDS, ensuring greater homogeneity in the study population and diagnostic criteria compared to earlier meta-analyses [9,10]. In addition, the inclusion of studies performed during the COVID-19 pandemic provides more contemporary data and extends the applicability of our findings across different ARDS causes. Our study also highlights the potential relevance of echocardiographic assessment of RV function in patients with moderate-to-severe ARDS, supporting its role in identifying patients at risk. However, further interventional studies are required to determine whether RV-targeted management, aimed at reducing RV afterload and optimizing RV–pulmonary artery coupling improves outcomes.

Several limitations should be acknowledged. First, only studies in English were included, which could introduce a potential source of selection bias. Nevertheless, as most reliable articles are in English, this bias is probably minimal. Second, in most studies, echocardiography was not performed prospectively on all consecutive ARDS patients. This could introduce a selection bias which might lead to an overestimation of both the prevalence of RV injury and its association with mortality. Third, we observed moderate to substantial clinical (patients, COVID-19 status, ARDS severity and inconsistently reported adjunctive therapies), methodological (study design, RV-injury definitions, timing and number of echocardiography examinations) and statistical (as illustrated by I^2^ values) heterogeneity across studies and analyses. Although the association between RV injury and mortality remained consistent across sensitivity analyses, this heterogeneity limits the precision and generalizability of the pooled estimates which should be considered as a global signal rather than a precise effect size. Fourth, visual inspection of the funnel plot suggested some asymmetry, and Egger’s regression test indicated statistical evidence of asymmetry. While this finding should be interpreted with caution, as the observed pattern was not restricted to small studies and may reflect underlying clinical and methodological heterogeneity, these results suggest the presence of publication bias. Fifth, key markers of ARDS severity, particularly the PaO₂/FiO₂ ratio, were inconsistently reported across studies and rarely available separately for patients with and without RV injury. A severity-stratified meta-regression to accurately assess the impact of ARDS severity on RV injury occurrence was therefore not feasible and this point deserves future individual-patient-data meta-analysis. Sixth, data on LV function were rarely reported across studies, which prevented to evaluate the role of LV dysfunction in RV injury. Seventh, it cannot be excluded that the results of our analysis according to COVID-19 status may have been affected by changes in ARDS management and echocardiographic practice in COVID-19 patients over time. Finally, studies relied exclusively on echocardiographic definitions of RV injury. It cannot be excluded that different results would be observed using hemodynamic definitions, such as those derived from pulmonary artery catheterization, which remains the reference method for assessing right heart function and pulmonary vascular load. Furthermore, most studies lacked concomitant hemodynamic parameters from echocardiographic examinations, which prevented an assessment of the relationship between RV injury and the severity of hemodynamic disorders in patients, as well as the relationship between RV injury and septic shock, which is the leading cause of mortality in patients with ARDS.

## Conclusions

In patients with ARDS receiving protective ventilation, RV injury is frequent and is consistently associated with mortality across heterogeneous definitions and settings. Further studies enrolling consecutive patients, with standardized and repeated echocardiographic assessment of RV function, are needed to establish causal inference.

## Author's contributions

MJ, AVB et PV conceived and designed the study. MJ, AJ, AM, MP, PV and BE collected data. MJ, AJ, AM, MP, AVB, PV and BE analyzed and interpreted the data. MJ and BE drafted the first version of the manuscript report. All authors contributed drafting the manuscript and approved the final version of the manuscript.

## Ethics approval

Not applicable. All studies have been independently reviewed and approved by the local Institutional Review Board. The protocol of this meta-analysis has been registered on PROSPERO (CRD42024568063).

## Funding

No funding.

## Consent to participate

Not applicable.

## Consent for publication

Not applicable.

## Availability of data and material

The datasets used and/or analysed during the current study are available from the corresponding author on reasonable request.

## Declaration of competing interest

The authors have no competing interests to declare.
