# Supplementary material for: Prevalence and prognosis impact of right ventricular injury in patients with acute respiratory distress syndrome: a systematic review and meta-analysis
Source: Ann Intensive Care. 2026 Jul 16;16:100117. doi: 10.1016/j.aicoj.2026.100117 (PMC13452399; doi:10.1016/j.aicoj.2026.100117)
Supplement: Supplementary file 1 [file mmc1.docx]

**Prevalence and prognosis impact of right ventricular injury in patients with acute respiratory distress syndrome: a systematic review and meta-analysis**

Mathieu JOZWIAK^1,2^, Adrien JOSEPH^3,4^, Alan MOUROUGAYEN^1^, Matthieu PETIT^3,4^, Antoine VIEILLARD-BARON^3,4^, Philippe VIGNON^5,6,7^ and Bruno EVRARD^5,6,7^

^1^ Service de Médecine Intensive Réanimation, CHU de Nice, Nice, France

^2^ UR2CA, Equipe CARRES, Université Côte d'Azur, Nice, France

^3^ Medical and surgical ICU, University hospital Ambroise Pare, AP-HP, Boulogne-Billancourt, France

^4^ UMR 1018, CESP, Université Versailles Saint Quentin en Yveline, France

^5^ Medical Surgical Intensive Care Unit, Limoges University Hospital, Limoges, France

^6^ CIC 1435-INSERM, Limoges, France

^7^ University of Limoges, Limoges, France

***Corresponding author***

Mathieu JOZWIAK, MD PhD

Service de Médecine Intensive Réanimation, CHU de Nice

151 route Saint Antoine de Ginestière

06200 Nice, France

[jozwiak.m@chu-nice.fr](mailto:jozwiak.m@chu-nice.fr)

**Supplemental figures legend**

**Figure S1 : Sensitivity analyses showing unadjusted (panel A) and adjusted (panels B and C) pooled mortality according to the COVID-19 status.**

OR: odds ratio, HR: hazard ratio, CI: confidence interval

**Figure S2 : Sensitivity analysis showing unadjusted (panel A) and adjusted (panels B and C) mortality after excluding studies including patients under venovenous extracorporeal membrane oxygenation.**

OR: odds ratio, HR: hazard ratio, CI: confidence interval

**Figure S3 : Sensitivity analysis showing unadjusted (panel A) and adjusted (panels B and C) mortality after excluding studies of moderate and low-quality according to the Newcastle-Ottawa quality assessment scale (NOS score <8).**

OR: odds ratio, HR: hazard ratio, CI: confidence interval

**Figure S4 : Funnel plot for unadjusted mortality.**

**Figure S1**

**Figure S2**

**Figure S3**

**Figure S4**

**Table S1. PRISMA 2020 Checklist for reporting systematic reviews and meta-analyses.**

| **Section and Topic** | **Item #** | **Checklist item** | **Location where item is reported** |
| --- | --- | --- | --- |
| **TITLE** | | |  |
| Title | 1 | Identify the report as a systematic review. | P1 |
| **ABSTRACT** | | |  |
| Abstract | 2 | See the PRISMA 2020 for Abstracts checklist. | P2 |
| **INTRODUCTION** | | |  |
| Rationale | 3 | Describe the rationale for the review in the context of existing knowledge. | P5 |
| Objectives | 4 | Provide an explicit statement of the objective(s) or question(s) the review addresses. | P5 |
| **METHODS** | | |  |
| Eligibility criteria | 5 | Specify the inclusion and exclusion criteria for the review and how studies were grouped for the syntheses. | P6 & P7 |
| Information sources | 6 | Specify all databases, registers, websites, organisations, reference lists and other sources searched or consulted to identify studies. Specify the date when each source was last searched or consulted. | P6 |
| Search strategy | 7 | Present the full search strategies for all databases, registers and websites, including any filters and limits used. | P6 |
| Selection process | 8 | Specify the methods used to decide whether a study met the inclusion criteria of the review, including how many reviewers screened each record and each report retrieved, whether they worked independently, and if applicable, details of automation tools used in the process. | P7 |
| Data collection process | 9 | Specify the methods used to collect data from reports, including how many reviewers collected data from each report, whether they worked independently, any processes for obtaining or confirming data from study investigators, and if applicable, details of automation tools used in the process. | P7 |
| Data items | 10a | List and define all outcomes for which data were sought. Specify whether all results that were compatible with each outcome domain in each study were sought (e.g. for all measures, time points, analyses), and if not, the methods used to decide which results to collect. | P7 |
|  | 10b | List and define all other variables for which data were sought (e.g. participant and intervention characteristics, funding sources). Describe any assumptions made about any missing or unclear information. | P7 |
| Study risk of bias assessment | 11 | Specify the methods used to assess risk of bias in the included studies, including details of the tool(s) used, how many reviewers assessed each study and whether they worked independently, and if applicable, details of automation tools used in the process. | P7 & P8 |
| Effect measures | 12 | Specify for each outcome the effect measure(s) (e.g. risk ratio, mean difference) used in the synthesis or presentation of results. | P8 & P9 |
| Synthesis methods | 13a | Describe the processes used to decide which studies were eligible for each synthesis (e.g. tabulating the study intervention characteristics and comparing against the planned groups for each synthesis (item #5)). | P8 & P9 |
|  | 13b | Describe any methods required to prepare the data for presentation or synthesis, such as handling of missing summary statistics, or data conversions. | P8 & P9 |
|  | 13c | Describe any methods used to tabulate or visually display results of individual studies and syntheses. | P8 & P9 |
|  | 13d | Describe any methods used to synthesize results and provide a rationale for the choice(s). If meta-analysis was performed, describe the model(s), method(s) to identify the presence and extent of statistical heterogeneity, and software package(s) used. | P8 & P9 |
|  | 13e | Describe any methods used to explore possible causes of heterogeneity among study results (e.g. subgroup analysis, meta-regression). | P8 & P9 |
|  | 13f | Describe any sensitivity analyses conducted to assess robustness of the synthesized results. | P8 & P9 |
| Reporting bias assessment | 14 | Describe any methods used to assess risk of bias due to missing results in a synthesis (arising from reporting biases). | P8 & P9 |
| Certainty assessment | 15 | Describe any methods used to assess certainty (or confidence) in the body of evidence for an outcome. | P8 & P9 |
| **RESULTS** | | |  |
| Study selection | 16a | Describe the results of the search and selection process, from the number of records identified in the search to the number of studies included in the review, ideally using a flow diagram. | P10 |
|  | 16b | Cite studies that might appear to meet the inclusion criteria, but which were excluded, and explain why they were excluded. | P10 |
| Study characteristics | 17 | Cite each included study and present its characteristics. | P10 |
| Risk of bias in studies | 18 | Present assessments of risk of bias for each included study. | P12 |
| Results of individual studies | 19 | For all outcomes, present, for each study: (a) summary statistics for each group (where appropriate) and (b) an effect estimate and its precision (e.g. confidence/credible interval), ideally using structured tables or plots. | P10 & P11 |
| Results of syntheses | 20a | For each synthesis, briefly summarise the characteristics and risk of bias among contributing studies. | P10 & P11 |
|  | 20b | Present results of all statistical syntheses conducted. If meta-analysis was done, present for each the summary estimate and its precision (e.g. confidence/credible interval) and measures of statistical heterogeneity. If comparing groups, describe the direction of the effect. | P10 & P11 |
|  | 20c | Present results of all investigations of possible causes of heterogeneity among study results. | P11 |
|  | 20d | Present results of all sensitivity analyses conducted to assess the robustness of the synthesized results. | P11 |
| Reporting biases | 21 | Present assessments of risk of bias due to missing results (arising from reporting biases) for each synthesis assessed. | P10 & P11 |
| Certainty of evidence | 22 | Present assessments of certainty (or confidence) in the body of evidence for each outcome assessed. | P10 & P11 |
| **DISCUSSION** | | |  |
| Discussion | 23a | Provide a general interpretation of the results in the context of other evidence. | P13-15 |
|  | 23b | Discuss any limitations of the evidence included in the review. | P16 & P17 |
|  | 23c | Discuss any limitations of the review processes used. | P16 & P17 |
|  | 23d | Discuss implications of the results for practice, policy, and future research. | P16 & P17 |
| **OTHER INFORMATION** | | |  |
| Registration and protocol | 24a | Provide registration information for the review, including register name and registration number, or state that the review was not registered. | P2 |
|  | 24b | Indicate where the review protocol can be accessed, or state that a protocol was not prepared. | P2 |
|  | 24c | Describe and explain any amendments to information provided at registration or in the protocol. | - |
| Support | 25 | Describe sources of financial or non-financial support for the review, and the role of the funders or sponsors in the review. | P4 |
| Competing interests | 26 | Declare any competing interests of review authors. | P4 |
| Availability of data, code and other materials | 27 | Report which of the following are publicly available and where they can be found: template data collection forms; data extracted from included studies; data used for all analyses; analytic code; any other materials used in the review. | P6 |

**Table S2: Multivariate analyses used in the 23 studies included in the meta-analysis.**

| # | Studies | Multivariate analysis | Statistical model | Outcome | Variables of interest | Main variables considered for adjustment |
| --- | --- | --- | --- | --- | --- | --- |
| 1 | Beyls 2022 (18) | Yes | Cox model | Day-30 mortality | RV systolic dysfunction | SAPS II, ACP |
| 2 | Boissier 2013 (7) | Yes | Logistic regression | Day-28 mortality / ACP occurrence | ACP | McCabe-Jackson class, non-pulmonary ARDS, driving pressure, lactic acidosis. |
| 3 | Cain 2024 (11) | Yes | Logistic regression | Death | RV dysfunction | LV dysfonction V |
| 4 | Cheong 2024 (24) | Yes | Logistic regression | In-hospital mortality | RV basal diameter and ACP | Age, APACHE II. |
| 5 | Chotalia 2022 (19) | Yes | Logistic regression | Day-90 mortality | RV dysfunction phenotypes | Age, respiratory compliance PEEP, peak pressure, PaO_2_/FiO_2_ ratio, PaCO_2_, pH, radiologic condensations, RRT, vasopressors dose. |
| 6 | Chotalia 2021 (17) | No | - | - | - | - |
| 7 | Evrard 2023 (12) | Yes | Cox model | ICU mortality | PSM / ACP | No clinical variable |
| 8 | Huang 2023 (13) | Yes | Accelerated failure time and multistate model | ICU mortality | RV dysfunction phenotypes | No clinical variable |
| 9 | Jozwiak 2024 (14) | Yes | Cox model | Day-28 mortality | RV injury | Intubation, PEEP, driving pressure, PaO_2_/FiO_2_ ratio, PaCO_2_, norepinephrine (administration + dose) |
| 10 | Khorsandi 2022 (20) | No | - | - | - | - |
| 11 | Lazzeri 2023 (23) | Yes | Logistic regression | ICU mortality | RV dilatation and dysfonction | Age, SOFA score, duration from onset of symptoms, duration of mechanical ventilation before ECMO ≥10 days, serum creatinine |
| 12 | Lazzeri 2018 (34) | Yes | Logistic regression | ICU mortality | RV dilatation | BMI, lactate |
| 13 | Lazzeri 2018 (35) | Yes | Logistic regression | ICU mortality | RV dilatation | Vasopressors before ECMO, BMI, lactate, PaO_2_ |
| 14 | Lhéritier 2013 (31) | Yes | Logistic regression | Day-28 mortality / ACP occurrence | ACP | Plateau pressure, PaCO_2_ |
| 15 | McCall 2022 (21) | Yes | Logistic regression | Day-30 mortality | RV dysfunction | Age, biological sex, ethnic group |
| 16 | Mekontso Dessap 2016 (8) | Yes | Logistic regression | ACP occurrence | ACP | Biological sex, age, SAPS II, shock, PaO_2_/FiO_2_ratio, driving pressure, prone positioning. |
| 17 | Petit 2024 (25) | Yes | Cox model | Day-90 mortality | RV injury | Age, SOFA score, immunosuppression, steroids administration, driving pressure, prone positioning |
| 18 | Lazzeri 2016 (32) | Yes | Logistic regression | ICU mortality | RV dilatation | TAPSE<16 mm, BMI, RVEDA/LVEDA |
| 19 | See 2017 (33) | Yes | Logistic regression | ICU mortality | ACP | Age, APACHE II |
| 20 | Tsolaki 2024 (15) | Yes | Logistic regression | Survival | RV and LV systolic function | D-Dimer, urea, troponin, respiratory compliance, plateau pressure, driving pressure, RVEDV, RVESV, pericardial effusion, LV longitudinal strain, RV longitudinal strain, PASP, VTI_RVOT_ |
| 21 | Valenzuela 2022 (22) | Yes | Logistic regression | ICU mortality | ACP | APACHE II, SOFA score, Heart rate, Norepinephrine, lactate, CRT, ScvO_2_, peak pressure, driving pressure, respiratory rate, FiO_2_, PaO_2_/FiO_2_ ratio, prone positioning, pH, PaCO_2_, troponin, D-Dimer, Mitral E wave, LVOT-VTI, TAPSE, LVEF |
| 22 | Xingzheng 2024 (37) | Yes | Logistic regression | ACP occurrence | PEEP level | BMI>25, pneumonia, pH<7.35, PaCO_2_>45 mmHg |
| 23 | Zeiton 2018 (36) | No | - | - | - | - |

ACP: Acute cor pulmonale, APACHE : acute physiology and chronic health evaluation, ARDS : acute respiratory distress syndrome, BMI : body mass index, CRT : capillary refill time, ECMO: extracorporeal membrane oxygenation, FiO_2_: inspired fraction of oxygen, LV : left ventricular, LVEDA : left ventricular end-diastolic area, LVEF : left ventricular ejection fraction, LVOT: left ventricular outflow tract, PaO_2_: partial arterial pressure of oxygen, PaCO_2_: partial arterial pressure of carbon dioxide, PASP: pulmonary artery systolic pressure, PEEP: positive end-expiratory pressure, PSM : paradoxical septal motion, RRT: renal replacement therapy, RV: right ventricular, RVEDA: right ventricular end-diastolic area, RVEDV: right ventricular end-diastolic volume, RVESV: right ventricular end-systolic volume, RVOT: right ventricular outflow tract, ScvO_2_: central venous oxygen saturation, SOFA : sepsis-related organ failure assessment, TAPSE: tricuspid annular plane systolic excursion, VTI: velocity-time integral

**Table S3: Mortality endpoints and patient outcomes in the 23 studies included in the meta-analysis.**

| **Study** | **Year** | **Sample**  **size** | **Mortality endpoint** | **Mortality**  **(%)** | **Prone positioning (%)** | **VV ECMO (%)** | **Inhaled NO**  **(%)** | **RRT**  **(%)** | **ICU length of stay**  **(mean days)** |
| --- | --- | --- | --- | --- | --- | --- | --- | --- | --- |
| Boissier (7) | 2013 | 226 | ICU | 50 | 31 | NA | 26 | NA | 31 |
| Lheritier (31) | 2013 | 200 | 28-day | 23 | 35 | NA | 13 | 23 | NA |
| Lazzeri (32) | 2016 | 74 | ICU | 42 | NA | 0* | NA | NA | 24 |
| Mekontso-Dessap (8) | 2016 | 752 | In-hospital | 43 | 29 | NA | NA | NA | NA |
| Lazzeri (34) | 2018 | 121 | ICU | 26 | NA | NA | NA | NA | 7 |
| See (33) | 2017 | 234 | ICU | 43 | NA | 0* | NA | NA | 25 |
| Lazzeri (35) | 2018 | 92 | ICU | 43 | 11 | 0* | NA | NA | 19 |
| Zeiton (36) | 2018 | 45 | 28-day | 67 | NA | NA | NA | NA | NA |
| Chotalia (17) | 2021 | 172 | 90-day | 41 | 67 | NA | NA | 47 | NA |
| Beyls (18) | 2022 | 86 | 28-day | 22 | NA | 17 | 62 | 29 | 25 |
| Chotalia (19) | 2022 | 305 | 90-day | 37 | 72 | NA | NA | 51 | NA |
| Khorsandi (20) | 2022 | 33 | ICU | 39 | NA | 100 | NA | NA | NA |
| McCall (21) | 2022 | 112 | 28-day | 47 | 49 | 13 | NA | 25 | NA |
| Valenzuela (22) | 2022 | 140 | ICU | 29 | 46 | NA | NA | NA | NA |
| Evrard (12) | 2023 | 182 | ICU | 36 | NA | 0 | NA | NA | 19 |
| Huang (13) | 2023 | 281 | ICU | 54 | NA | 100 | NA | 48 | NA |
| Lazzeri (23) | 2023 | 61 | ICU | 31 | 35 | NA | 0 | 1 | NA |
| Cain (11) | 2024 | 243 | ICU | 39 | NA | 52 | 41 | NA | 36 |
| Cheong (24) | 2024 | 114 | In-hospital | 36 | 58 | NA | NA | 7 | 21 |
| Jozwiak (14) | 2024 | 118 | ICU | 19 | 74 | 3 | 45 | 20 | NA |
| Petit (25) | 2024 | 158 | 90-day | 49 | NA | NA | NA | NA | NA |
| Tsolaki (15) | 2024 | 176 | ICU | 68 | NA | NA | NA | NA | NA |
| Xingzheng (37) | 2024 | 52 | ICU | 19 | NA | NA | NA | NA | 12 |

ICU: intensive care unit, NA: non-available, NO: nitric oxide, RRT: renal replacement therapy, VV-ECMO: venovenous extracorporeal membrane oxygenation.

* VV-ECMO pre-implantation

# **Table S4. Quality assessment of the studies according to Newcastle-Ottawa quality assessment scale for cohort studies.**

| # | Studies | SELECTION (4★) | | | | COMPARABILITY (2★) | | OUTCOMES (3★) | | | TOTAL |
| --- | --- | --- | --- | --- | --- | --- | --- | --- | --- | --- | --- |
|  |  | Representativeness  of exposed cohort | Selection of  non-exposed cohort | Ascertainment of exposure | Outcome of interest  not present at  the start of the study | Comparability for the most important factor  (ARDS severity) | Comparability for any additional factors  (age and comorbidities) | Assessment of outcomes | Length of  follow-up | Adequacy  of follow-up |  |
| 1 | Beyls 2022 (18) | — | **★** | **★** | **★** | **★** | **★** | **★** | **★** | — | **7/9** |
| 2 | Boissier 2013 (7) | **★** | **★** | **★** | **★** | **★** | **★** | **★** | **★** | **★** | **9/9** |
| 3 | Cain 2024 (11) | — | — | **★** | **★** | **★** | — | **★** | **★** | — | **5/9** |
| 4 | Cheong 2024 (24) | — | — | **★** | **★** | **★** | — | **★** | **★** | — | **5/9** |
| 5 | Chotalia 2022 (19) | — | — | **★** | **★** | **★** | — | **★** | **★** | — | **5/9** |
| 6 | Chotalia 2021 (17) | — | — | **★** | **★** | **★** | **★** | **★** | **★** | — | **6/9** |
| 7 | Evrard 2023 (12) | **★** | **★** | **★** | **★** | **★** | **★** | **★** | **★** | **★** | **9/9** |
| 8 | Huang 2023 (13) | — | **★** | **★** | **★** | **★** | **★** | **★** | **★** | — | **7/9** |
| 9 | Jozwiak 2024 (14) | **★** | **★** | **★** | **★** | **★** | **★** | **★** | **★** | **★** | **9/9** |
| 10 | Khorsandi 2022 (20) | — | — | **★** | — | **★** | — | **★** | **★** | — | **4/9** |
| 11 | Lazzeri 2023 (23) | **★** | **★** | **★** | **★** | **★** | **★** | **★** | **★** | — | **8/9** |
| 12 | Lazzeri 2018 (34) | **★** | **★** | **★** | **★** | **★** | **★** | **★** | **★** | — | **8/9** |
| 13 | Lazzeri 2018 (35) | **★** | **★** | **★** | **★** | **★** | **★** | **★** | **★** | — | **8/9** |
| 14 | Lhéritier 2013 (31) | **★** | **★** | **★** | **★** | **★** | **★** | **★** | **★** | **★** | **9/9** |
| 15 | McCall 2022 (21) | **★** | **★** | **★** | **★** | **★** | **★** | **★** | **★** | — | **8/9** |
| 16 | Mekontso Dessap 2016 (8) | **★** | **★** | **★** | **★** | **★** | **★** | **★** | **★** | **★** | **9/9** |
| 17 | Petit 2024 (25) | — | — | **★** | **★** | **★** | — | **★** | **★** | — | **5/9** |
| 18 | Lazzeri 2016 (32) | **★** | **★** | **★** | **★** | — | **★** | **★** | **★** | — | **7/9** |
| 19 | See 2017 (33) | **★** | **★** | **★** | **★** | **★** | **★** | **★** | **★** | — | **8/9** |
| 20 | Tsolaki 2024 (15) | **★** | **★** | **★** | **★** | **★** | **★** | **★** | **★** | — | **8/9** |
| 21 | Valenzuela 2022 (22) | **★** | **★** | **★** | **★** | **★** | **★** | **★** | **★** | **★** | **9/9** |
| 22 | Xingzheng 2024 (37) | **★** | **★** | **★** | **★** | **★** | **★** | **★** | **★** | **★** | **9/9** |
| 23 | Zeiton 2018 (36) | **★** | **★** | **★** | **★** | — | — | **★** | **★** | — | **6/9** |

Total score of 8 or 9/9 : high quality, total score of 6 or 7/9 : moderate quality, total score < 6/9 : low quality

# **Table S5. Certainty assessment according to GRADE classification.**

| **Outcomes** | **Number of studies** | **Study**  **design** | **Risk**  **of bias** | **Inconsistency** | **Indirectness** | **Imprecision** | **Publication bias** | **Large**  **effect** | **Dose response** | **All plausible confounding and bias** | **Certainty** |
| --- | --- | --- | --- | --- | --- | --- | --- | --- | --- | --- | --- |
| **PRIMARY OUTCOME** | | | | | | | | | | | |
| ICU mortality (unadjusted analyses) | 11 | Observational | Serious | Not serious | Not serious | Serious | Not serious | Serious | NA | Serious | **⊕⊕○○ Low** |
| ICU mortality (adjusted analyses) | 11 | Observational | Serious | Not serious | Not serious | Serious | Not serious | Serious | NA | Serious | **⊕⊕○○ Low** |
| 28-day mortality | 4 | Observational | Serious | Not serious | Not serious | Very serious | Serious | Not serious | NA | Serious | **⊕○○○ Very Low** |
| 90-day mortality | 3 | Observational | Serious | Not serious | Not serious | Very serious | Serious | Not serious | NA | Serious | **⊕○○○ Very Low** |
| In-hospital mortality | 2 | Observational | Serious | Not serious | Not serious | Very serious | Serious | Not serious | NA | Serious | **⊕○○○ Very Low** |
| **SECONDARY OUTCOMES** | | | | | | | | | | | |
| Prevalence of RV injury | 23 | Observational | Serious | Serious | Not serious | Serious | Not serious | NA | NA | Serious | **⊕○○○ Very Low** |
| ICU mortality according to COVID-19 status | 19 | Observational | Serious | Not serious | Not serious | Serious | Not serious | Serious | NA | Serious | **⊕⊕○○ Low** |
| ICU mortality with RV injury defined as ACP | 13 | Observational | Serious | Serious | Not serious | Serious | Not serious | Serious | NA | Serious | **⊕○○○ Very Low** |
| ICU mortality with RV injury defined as RV systolic dyfunction | 4 | Observational | Serious | Not serious | Not serious | Serious | Not serious | Serious | NA | Serious | **⊕⊕○○ Low** |
| ICU mortality after excluding patients requiring VV-ECMO | 11 | Observational | Serious | Not serious | Not serious | Serious | Not serious | Serious | NA | Serious | **⊕⊕○○ Low** |

ACP : acute cor pulmonale, ICU : intensive care unit, NA : not applicable, RV : right ventricular, VV-ECMO : venovenous extracorporeal membrane oxygenation
